# Polylactide, Processed by a Foaming Method Using Compressed Freon R134a, for Tissue Engineering

**DOI:** 10.3390/polym13203453

**Published:** 2021-10-09

**Authors:** María Aguado, Laura Saldaña, Eduardo Pérez del Río, Judith Guasch, Marc Parera, Alba Córdoba, Joaquín Seras-Franzoso, Olivia Cano-Garrido, Esther Vázquez, Antonio Villaverde, Jaume Veciana, Imma Ratera, Nuria Vilaboa, Nora Ventosa

**Affiliations:** 1 Institut de Ciència de Materials de Barcelona, ICMAB-CSIC, Campus UAB, 08193 Bellaterra, Spain; m.aguado.olalla@gmail.com (M.A.); eperez2@icmab.es (E.P.d.R.); jguasch@icmab.es (J.G.); marc.parera@applus.com (M.P.); acordoba@nanomol-tech.com (A.C.); vecianaj@icmab.es (J.V.); 2 CIBER de Bioingeniería, Biomateriales y Nanomedicina (CIBER-BBN), 28029 Madrid, Spain; laura.saldana@salud.madrid.org (L.S.); joaquin.seras@gmail.com (J.S.-F.); olivia.cano.garrido@gmail.com (O.C.-G.); Esther.Vazquez@uab.cat (E.V.); antoni.villaverde@uab.cat (A.V.); 3 Hospital Universitario La Paz-IdiPAZ, Paseo de la Castellana 261, 28046 Madrid, Spain; 4 Dynamic Biomimetics for Cancer Immunotherapy, Max Planck Partner Group, ICMAB-CSIC, Campus UAB, Bellaterra, 08193 Barcelona, Spain; 5 Institut de Biotecnologia i Biomedicina, Universitat Autònoma de Barcelona, Bellaterra, 08193 Barcelona, Spain; 6 Departament de Genètica i de Microbiologia, Universitat Autònoma de Barcelona, Bellaterra, 08193 Barcelona, Spain

**Keywords:** 3D scaffolds, biomaterial engineering, tissue engineering, mesenchymal stem cells, polymeric foams, surface functionalization, protein nanoparticles, cell growth, compressed fluids, Freon R134a

## Abstract

Fabricating polymeric scaffolds using cost-effective manufacturing processes is still challenging. Gas foaming techniques using supercritical carbon dioxide (scCO_2_) have attracted attention for producing synthetic polymer matrices; however, the high-pressure requirements are often a technological barrier for its widespread use. Compressed 1,1,1,2-tetrafluoroethane, known as Freon R134a, offers advantages over CO_2_ in manufacturing processes in terms of lower pressure and temperature conditions and the use of low-cost equipment. Here, we report for the first time the use of Freon R134a for generating porous polymer matrices, specifically polylactide (PLA). PLA scaffolds processed with Freon R134a exhibited larger pore sizes, and total porosity, and appropriate mechanical properties compared with those achieved by scCO_2_ processing. PLGA scaffolds processed with Freon R134a were highly porous and showed a relatively fragile structure. Human mesenchymal stem cells (MSCs) attached to PLA scaffolds processed with Freon R134a, and their metabolic activity increased during culturing. In addition, MSCs displayed spread morphology on the PLA scaffolds processed with Freon R134a, with a well-organized actin cytoskeleton and a dense matrix of fibronectin fibrils. Functionalization of Freon R134a-processed PLA scaffolds with protein nanoparticles, used as bioactive factors, enhanced the scaffolds’ cytocompatibility. These findings indicate that gas foaming using compressed Freon R134a could represent a cost-effective and environmentally friendly fabrication technology to produce polymeric scaffolds for tissue engineering approaches.

## 1. Introduction

Scaffolds for tissue engineering should be degradable and biocompatible and have an appropriate porous structure and mechanical properties to allow cell colonization and growth [1,2,3,4,5]. Indeed, the presence of pores is essential not only for allowing cell migration and growth but also for enabling the diffusion of nutrients, oxygen and metabolic waste, which are necessary for the tissue regeneration process. Although cell migration and nutrient transport are ensured with the presence of intercommunicated pores of approximately 150 μm in diameter, larger sizes (>300 μm) are recommended to promote vascularization and enhance osteogenesis [2]. However, a high degree of porosity usually implies a reduction in the mechanical properties of scaffolds, which could compromise the structure’s integrity [4,5,6]. Consequently, a compromise between the pores’ structure and dimensions and the mechanical properties is required.

Developing cost-effective procedures to fabricate synthetic polymers with controlled porous structures is an ongoing challenge in the biomedical engineering field. A variety of methods are available, including conventional solvent casting/particulate leaching, thermally induced phase separation, freeze drying, compression molding, electrospinning, as well as more advanced processing and fabrication methods such as those based on 3D printing [2,7,8]. However, all such conventional methods entail the use of organic solvents and high temperatures during the fabrication process, which limit their use when loading bioactive molecules into the matrices. Indeed, growth factors and other proteins are prone to denaturation at high temperatures or in the presence of certain organic solvents. In addition, solvent residues can trigger undesired harmful side effects at the implantation site. Salt leaching with sodium chloride is a common strategy for obtaining porous scaffolds; however, pore size distributions are difficult to reproduce, and therefore the scale-up of this procedure is highly challenging. Moreover, additive manufacturing methods such as rapid prototyping, which enables the generation of patient-customized, precise, and complex architectures, face several scale-up and cost-related difficulties that limit their massive clinical application. 

An attractive alternative for overcoming the limitations associated with conventional methods is the “gas foaming” technique, which uses supercritical carbon dioxide (scCO_2_) to obtain materials with a high degree of porosity (up to 80%) [9,10,11]. This organic solvent-free process can occur at physiological temperatures, allowing the incorporation of biological agents [12,13,14,15,16]. scCO_2_ is an attractive solvent because it is non-toxic, non-flammable and relatively inert [5,17]. Foaming with scCO_2_ requires pressures above the critical value (around 10 MPa) to achieve the gas’ supercritical state. To achieve these processing conditions (Tc of 304 K and Pc of 7.38 MPa), high-pressure equipment is required [18]. An alternative to scCO_2_ is 1,1,1,2-tetrafluoroethane, commercially known as Freon R134a or norflurane, which despite its higher price provides significant advantages over CO_2_ in supercritical processing, including a much lower pressure and temperature setting to become liquid (<2 MPa at room temperature), resulting not only in a reduction of the risks associated with working at high pressures but also in the cost of the equipment, with less specialized units and fittings, thereby facilitating its industrial application [19]. Similar to scCO_2_, Freon R134a is non-toxic and non-flammable, with insignificant ozone depletion potential compared with other freons, and it is currently used in numerous biomedical applications [20]. Freon R134a can be more easily compressed and recycled in a gas-foaming process than CO_2_ due to the lower Pc of Freon R134a. To our knowledge, however, its use for preparing polymeric scaffolds using the gas foaming technique has not been attempted to date. 

The functionalization of scaffold surfaces with bioactive factors has been investigated as a strategy to enhance cell colonization and growth [3,21,22,23]. Inclusion bodies like protein nanoparticles (pNPs) are deposits formed in bacteria due to recombinant protein overexpression [24]. These pNPs (in the range of few hundreds of nanometers) are commonly found in the bacteria cytoplasm [25]. In the past, such pNPs have been described as an obstacle in recombinant processes and considered as non-desired products. However, recent studies have demonstrated their potential as bioactive factors for tissue engineering. These protein aggregates not only are non-cytotoxic but, when incorporated to surfaces, also generate mechanical and biochemical signals that stimulate cell adhesion and proliferation [26,27,28,29,30,31,32]. The addition of bioactive factors to scaffolds can be achieved by various strategies, with direct surface adsorption being the most frequently used [4,21]. 

Due to their biocompatibility and biodegradability, polylactide (PLA) and poly(lactide-co-glycolide) (PLGA) saturated aliphatic polyesters stand out among the synthetic polymers used for tissue engineering [33,34,35,36,37,38]. In this study, we explored the use of compressed Freon R134a for processing PLA matrices at low pressure and compared the results with the same polymeric matrices processed at higher pressure using the well-implemented scCO_2_. For comparative purposes, we also fabricated PLGA matrices using compressed Freon R134a. To enhance their bioactivity, pNPs derived from green fluorescence protein (GFP), a commonly used marker for live-cell imaging, were used to functionalize the surface of the resulting porous scaffolds by means of a filtration process. Lastly, to explore the suitability of scaffolds processed with compressed fluids and functionalized with pNPs for bone tissue engineering applications, we investigated their cytocompatibility using human mesenchymal stem cells (MSCs) as precursors of osteoblasts, the bone-forming cells.

## 2. Materials and Methods

### 2.1. Materials

We employed semicrystalline polylactide (PDL, LLA; abbreviated as PLA) and amorphous PLGA with different molecular weights and inherent viscosities. The PDL, LLA (70:30, with inherent viscosity between 5.7–6.5 dL/g), Resomer^®^ LR 708 (molecular weight, 150,000 Da), and PLGA (50:50, with inherent viscosity between 0.32–0.44 dL/g), and RESOMER RG503 (molecular weight, 30,000 Da) were purchased from Evonik Röhm GmbH (Darmstadt, Germany). Carbon dioxide (purity 99.995%) and Freon R134a were supplied by Carburos Metálicos-Air products S.A. (Barcelona, Spain). 

### 2.2. Methods

#### 2.2.1. Preparation of Polymer Disks

The desired mass of polymer was weighed and placed in a special polytetrafluoroethylene mold with a diameter of 13 mm and formed by two detachable parts, allowing for disk removal after preparation. The polymer pellets were then compressed with 3 tonnes for 20 s to form compressed non-porous polymer disks using a hydraulic press (Perkin Elmer, Waltham, MA, USA). Due to the lower diffusion of compressed fluids in semicrystalline polymers than in amorphous ones [9], an annealing pretreatment to amorphize the crystalline region of PLA was performed. As shown by the thermograms of the differential scanning calorimetry (DSC) (PerkinElmer DSC 8500 Lab System) (Supplementary Materials, Figures S1 and S2), the PLA crystalline phase was removed by thermal annealing of the disks in an oven at 150 °C. Fifteen minutes were sufficient for the complete amorphization of the PLA prior to its processing with compressed fluids. DSC analysis of the annealed PLA was performed from 20 °C to 250 °C at a heating rate of 10 °C/min. One mg of the sample was sealed into a 40-µL aluminum pan and heated under a nitrogen purge of 50 mL/min. the temperature calibrations were performed using indium as the standard.

#### 2.2.2. 3D Porous Scaffold Fabrication Using Compressed Fluids

**Figure 1:**
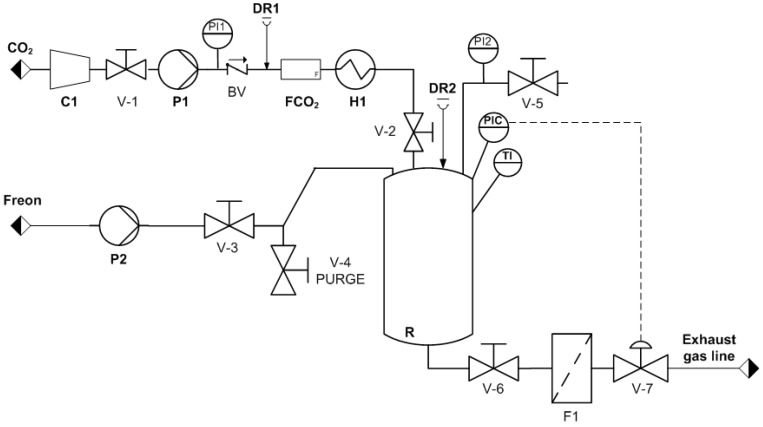
Diagram of the equipment used for preparing 3D porous polymeric scaffolds with compressed CO_2_ or Freon R134a. P1 and P2: pumps for compressing the fluids; DR1 and DR2: rupture disks; F1: mass flow meter; V-1/V-7: valves; H1: heater; R: high-pressure vessel; F1 and FCO_2_: filters; PI1 and PI2: pressure indicators; PIC: pressure controller; TI: temperature indicator.

We prepared 3D porous scaffolds from the polymer disks using a foaming process with compressed fluids in a high-pressure plant at a laboratory scale (Figure 1). Several polymer disks were placed in a high-pressure vessel using a special stainless-steel basket divided into seven levels. The vessel, R (300 mL), was pre-heated to the working temperature (Tw) and then pressurized to the desired pressure (Pw) by adding the corresponding compressed fluid (scCO_2_ or Freon R134a). A high-pressure pump (P1 or P2) was used to introduce the compressed fluid into the vessel up to the desired working pressure. The polymer/compressed-fluid mixture was maintained at Pw for a specific soaking time (ts). The vessel was then depressurized from Pw to ambient pressure at a constant flow rate by opening valve V-7 to 60% of its aperture. The foaming process occurs in three different steps. First, the polymer matrix is saturated with the corresponding compressed fluid at the working pressure and temperature. Second, once saturation is achieved, the diffusion of the compressed fluid forms a single phase of polymer/compressed fluid. Third, a decrease in pressure causes phase segregation, and the compressed fluid evaporates, leading to the generation of pores or foaming of the polymer. The resulting foamed polymeric matrices were then cut with a diamond saw to eliminate the generated outer non-porous layer and to shape the specimens as cylinders with a radius of 15 mm and thickness of 3.5 mm. Non-complete foaming of the polymer disks was detected visually by the non-expansion of the disk and by the presence of non-foamed polymer at the center of the disk, once it was cut with the diamond saw (Supplementary Materials, Figure S3). The resulting porous specimens were stored for 1 week at room temperature until no loss of weight (due to gas release) was registered, the specimens were then kept at 4 °C until use.

#### 2.2.3. Porous Scaffold Characterization

##### Solid Density and Porosity 

We calculated the density of the foamed (“apparent”) and unfoamed (“absolute”) scaffolds with a helium pycnometer (Ultrapycnometer 1200e, Quantachrome Instruments, Boynton Beach, FL, USA). For the foamed disks, the pycnometer determined the volume occupied by the solid material plus the volume of the closed porosity, while for the unfoamed disks, the device determined the volume of solid material without pores.

The porosity, including the closed and open porosity, was estimated by correlating the porosity (P) to the density (ρ) of the foamed and unfoamed materials [39]. We calculated the total porosity (P_T_) of the prepared materials by dividing the geometric density (ρ_geometric_) by the unfoamed density (ρ_unfoamed_), using the formula P_T_ = [1 − (ρ_geometric_/ρ_unfoamed_)]·100, where the geometric density (ρ_geometric_) refers to the density of the foamed disk, once the outer layer was removed, and using the foamed mass and geometric volume. We calculated this volume with the theoretical volume of a cylinder (v = π·r^2^·h, where r is the radius of the disk and h is the height). The diameter and height of each disk was measured using a standard caliper (Mitutoyo, Tokyo, Japan). Similarly, we calculated the closed porosity (P_closed_) by dividing the foamed density (ρ_pycnometry_) by the unfoamed density (ρ_unfoamed_), using the formula P_closed_ = (1 − ρ_pycnometry_/ρ_unfoamed_)·100. We then obtained the open porosity (P_O_) simply as the difference between the total and closed porosity (i.e, P_O_ = P_T_ − P_closed_).

We calculated the mean porosity values and corresponding standard deviations from the experimental measurements performed on three samples of each type of scaffold. 

##### Morphology by Scanning Electron Microscopy (SEM)

We analyzed the microscopic and nanoscopic morphologies of the porous polymeric matrices by scanning electron microscopy (Quanta 200 FEG-ESEM, FEI, Hillsboro, OR, USA). Prior to the analysis, the porous disks were coated with gold for 4 min at 20 mA in a sputter coater (K550X, Emitech, Surrey, UK) by modifying the inclination of the holder to achieve a homogeneous coverage of the scaffolds.

##### Micro X-ray Computed Tomography 

All scaffolds were characterized using X-ray micro-computed tomography (SkyScan-1272; Bruker, Kontich, Belgium), a non-destructive analysis in which the pore size distribution and 3D visualization can be simultaneously obtained. Cylindrical scaffolds with a thickness 3.5 mm and diameter of 15 mm were mounted in the equipment. Analyses were performed with a charged-couple device camera at a pixel size of 9 μm, using a source voltage of 50 kV and a current of 200 μA. All generated images were saved in TIFF format with a pixel size of 12 μm. We employed NRecon software (Micro Photonics, Allentown, PA, USA) to reconstruct cross-section images from the tomography projection images.

##### Rheological Properties

We measured the rheological properties of the porous scaffolds through the small-amplitude oscillatory shear technique, as previously shown [40,41,42], using a rheometer (HAAKE RheoStress RS600, Thermo Electron Corporation, Waltham, MA, USA) with a rotor diameter of 10 mm. This technique consists of applying a small-amplitude torsional oscillation that generates a shear flow on the sample. Strain and frequency sweeps were performed to determine the range of pressure and frequency where the scaffolds maintain their viscoelastic behavior and achieve the value of the shear modulus (G’).

#### 2.2.4. pNPs Production and Purification

pNPs were produced in the *E. coli* strain MC4100, transformed with the expression vector pTV1GFP. *E. coli* was grown in LB-rich medium supplemented with 100 μg/mL of ampicillin and 30 μg/mL of streptomycin at 37 °C and 250 rpm. Production of pNPs was induced when reaching an optical density at 550 nm of 0.5 by adding 1 mM isopropyl Beta-D-1-thiogalactopyranoside. After 3 h, the cell cultures were harvested for pNPs purification with a combination of mechanical and enzymatic procedures, as previously described [43]. 

#### 2.2.5. Porous Scaffold Functionalization with pNPs

Surface functionalization of the porous scaffolds was performed using a filtration procedure in which an aqueous suspension of pNPs was forced through the porous material. We first resuspended 600 μg of pNPs in 20 mL of phosphate-buffered saline (PBS) supplemented with a mixture of 1.6 mL of tetracycline, kanamycin and chloramphenicol to prevent microbial contamination of the scaffolds during their manipulation. The suspension was sonicated for 10 min, and 5 mL of the suspension was then filtered through the porous specimen to decorate the material. To increase the efficiency of the process, the procedure was repeated 3 times, each time using the same previously filtrated 5 mL. The pNPs-loaded scaffolds obtained were dried with compressed air and weighed before (m_0_) and after filtration and after the drying process (m_f_). The scaffolds were kept at −20 °C until use. pNPs loading was calculated using the following equation: (m_f_−m_0_)/m_0_.

We estimated the amount of pNPs retained on the surface of the functionalized scaffolds using a fluorescence spectrophotometer (Cary Eclipse Fluorescence Spectrometer, Santa Clara, CA, USA) by comparing the fluorescence intensities at 510 nm of the suspension before and after the decoration process. To study the penetrability of pNPs into PLA-based scaffolds, we cut a cross-section of the scaffold using a diamond wire saw and mounted it on glass-bottom slides (Nunc, Wiesbaden, Germany). Images were taken using a TCS SPE confocal microscope (Leica, Wetzlar, Germany). The fluorescence from pNPs was excited with a 488-nm laser line and collected at the emission range of 495–590 nm.

#### 2.2.6. Cell Culture and Viability Assay

Purified human bone marrow-derived MSCs (CD105^+^, CD29^+^, CD44^+^, CD14^−^, CD34^−^ and CD45^−^) were purchased from Lonza (Basel, Switzerland). The cells were cultured in a defined medium (Lonza) consisting of basal medium and SingleQuots growth supplements containing fetal bovine serum (FBS), L-glutamine, penicillin, and streptomycin. All experiments were performed below seven cell passages. The experiments were performed in duplicate using cells isolated from three different donors aged 20–31 years. Before cell seeding, scaffolds decorated or not with pNPs were incubated in DMEM containing 15% FBS and antibiotics (DMEM-15%FBS) for 24 h. We seeded 2 × 10^5^ MSCs on scaffolds placed in 24-well plates and cultured them in DMEM-15%FBS for 1–18 days. Cell viability was evaluated using the alamarBlue assay (Biosource, Nivelles, Belgium). Cells were incubated in DMEM containing 10% alamarBlue dye; 3 h later, the fluorescence emitted by the cell-reduced alamarBlue was quantified using a spectrofluorometer (Synergie4, Evry, France).

#### 2.2.7. Immunofluorescence Assays

The MSCs cultured for 8 days on the scaffolds were washed with PBS followed by fixation in 4% (*w*/*v*) paraformaldehyde in PBS and permeabilization with 0.1% Triton X-100 in PBS. For fibronectin immunostaining, the cells were blocked in 2% bovine serum albumin (BSA) in PBS containing 0.05% Tween 20 and then incubated with mouse anti-human fibronectin monoclonal antibody (Santa Cruz, Heidelberg, Germany) diluted 1:50 in 1% BSA in PBS. The cells were washed with 0.05% Tween 20 in PBS followed by incubation with goat anti-mouse Alexa-Fluor 488 (Molecular Probes, Leiden, The Netherlands) diluted 1:1000 in 1% BSA in PBS. To label actin cytoskeleton, the cells were stained with PBS containing 4 × 10^−7^ M phalloidin-TRITC (Sigma-Aldrich, Madrid, Spain). For nuclei staining, the cells were incubated in PBS containing 3 × 10^−6^ M 4,6-diamidino-2-phenylindole (DAPI; Sigma) before the confocal microscopy examination.

#### 2.2.8. Statistical Analysis

Experiments were conducted in duplicate, and the data are presented as mean values ± SD of four independent experiments. The statistical analyses were performed using the Statistical Package for the Social Sciences, version 15.0 (SPSS Inc., Chicago, IL, USA). The quantitative data were tested using the Kruskal-Wallis test followed by Dunn’s multiple comparison test, and the level of significance was set at p < 0.05. 

## 3. Results and Discussion

We studied the processing conditions of PLA with CO_2_ and Freon R134a compressed fluids and their influence on the physicochemical and mechanical properties of the resulting porous matrices. We also functionalized the surface of the obtained 3D porous matrices with bioactive pNPs and evaluated their in vitro cytocompatibility by culturing MSCs on them. Given that the bioactivity of 3D porous scaffolds depends not only on the fabrication routes and processing media but also on the nature of the material, we processed PLGA (a polymer widely used in the biomedical field) with Freon R134a.

### 3.1. Preparation of 3D Porous Scaffolds 

The three most common options for polymer foaming with compressed fluids are batch foaming, foam extrusion and the injection foam molding [9]. The methodology used in this study for preparing 3D porous scaffolds was based on batch foaming process, which is extensively employed in research because it allows for a fine control of the processing variables and is relatively simple to perform. However, extrusion foaming is the industrially preferred option for foaming due to its adaptability for continuous production and easier scaling-up potential [44].

**Figure 2:**
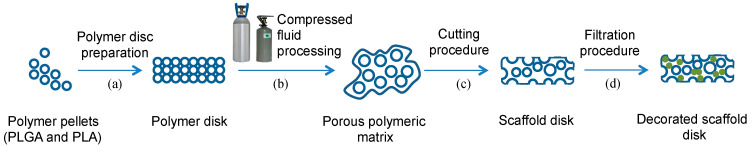
Scheme of the experimental procedure for preparing 3D porous scaffolds. (a) First, polymeric disks were prepared from PLA or PLGA polymer pellets. (b) The disks were then transformed into porous scaffolds using compressed fluid processing in a high-pressure plant. (c) The non-porous skin layer was cut out, obtaining a porous scaffold disk. (d) The disk was subsequently loaded with bioactive pNPs by a filtration procedure.

The general experimental procedure employed in this study to prepare the various types of porous polymeric matrices consisted of several steps (Figure 2). In a high-pressure vessel, polymer disks were exposed to compressed fluids (either Freon R134a or scCO_2_) at a given working pressure (Pw) and temperature (Tw) to ensure the compressed fluid reached a supercritical state for a suitable soaking time (t), depending on the polymer/compressed fluid mutual diffusivity, to allow sorption and fluid solubilization in the polymeric matrix. This was then followed by a depressurization step, which induced gas bubble nucleation and growth and the formation of the porous structure.

While scCO_2_ requires working pressures >10 MPa, Freon R134a requires almost an order of magnitude lower pressure (2 MPa) [45]. Although all resulting 3D scaffolds showed a porous structure, they also presented an outer non-porous layer. Outer layer formation, previously reported for the CO_2_ foaming process [16,46,47], has been attributed to rapid gas diffusivity as the gas escapes from the scaffold surface during depressurization and to the solubility of the CO_2_ at the pressure employed, the effects of which have been previously described [48]. The same mechanism could explain the formation of the outer non-porous layer in the case of the dense Freon. When depressurizing, the solubility of the Freon decreases, and, consequently, the diffusivity increases, resulting in the formation of this undesirable layer, which acts as a barrier for deep cell colonization and for the delivery of inductive factors to the inner part of the scaffold. We therefore developed a cutting procedure to remove the outer layer of the scaffold using a diamond saw, obtaining porous disks with the desired thickness of 3.5 mm. Lastly, the resulting 3D porous scaffolds were internally functionalized with controlled amounts of GFP-based pNPs. The gas foaming processing conditions evaluated for PLA and PLGA are shown in Table 1, which lists the operational process values for the pressure, temperature and soaking times that yielded complete foaming of the polymer disks and appropriate scaffold characteristics, such as a high degree of porosity and mechanical strength, to behave as scaffolds for tissue engineering. Foaming with Freon R134a required a slightly higher temperature than foaming with scCO_2_, as well as a longer soaking time.

Given that the molecular weight and viscosity of PLGA is lower than that of PLA, the solubility of dense Freon inside the polymer increases. Consequently, a lower Tw is needed to produce the expansion of PLGA disks inside the vessel. Indeed, a smaller mass of PLGA was employed to prepare the polymer disk due to the higher expansion during processing. It is known that the diffusion of compressed fluids is much lower in semicrystalline polymers than in amorphous ones, due to the free volume effect [9]. Therefore, the annealing pretreatment causing an amorphous transformation of the crystalline region of PLA was essential for achieving a complete polymer expansion by gas foaming. This pre-treatment was not needed for PLGA due to its amorphous nature.

### 3.2. 3D Porous Scaffold Characterization

Using SEM and micro-computed tomography, we characterized the morphology and pore size distribution of PLA processed with Freon R134a or scCO_2_ and of PLGA scaffolds processed with Freon R134a. 

**Figure 3:**
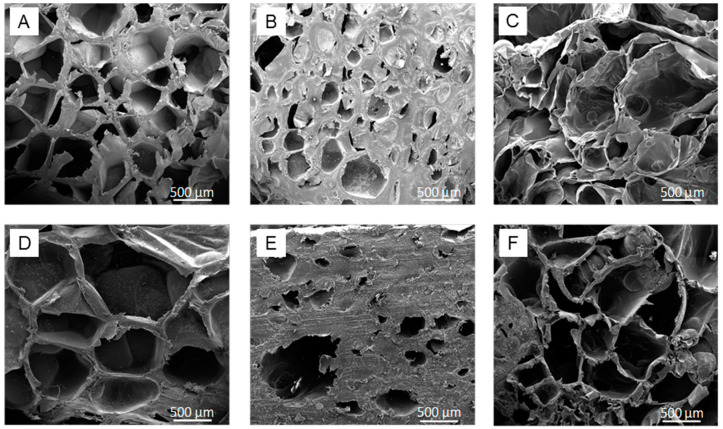
SEM images corresponding to longitudinal (**A**–**C**) and cross-sections (**D**–**F**) of (**A**,**D**) PLA processed with Freon R134a; (**B**,**E**) PLA processed with scCO_2_; and (**C**,**F**) PLGA processed with Freon R134a.

The SEM images of the longitudinal and cross-sections of the processed polymers revealed porous structures for the three materials (Figure 3), confirming that dense Freon can be used to successfully prepare 3D porous scaffolds of PLA and PLGA. Moreover, PLA-based scaffolds processed with Freon R134a showed larger pores with thinner walls than those processed with CO_2_.

**Figure 4:**
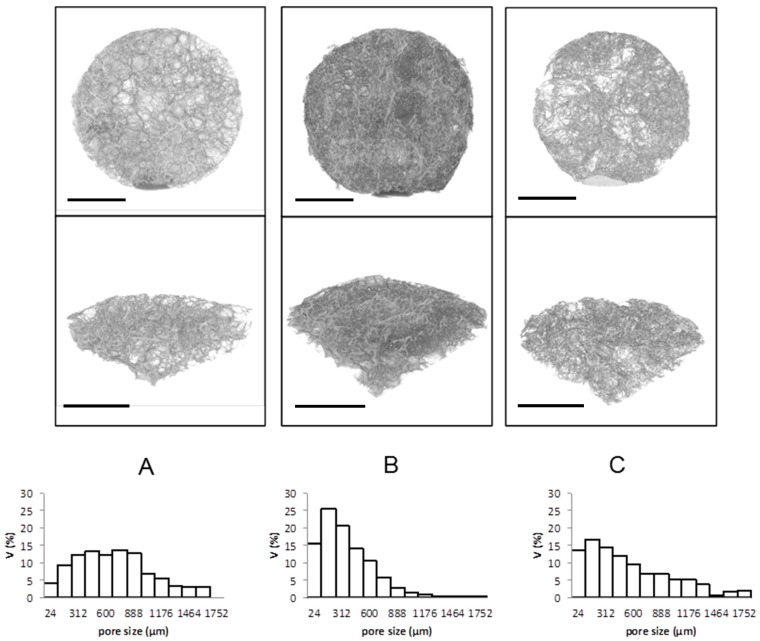
Top view and cross-section morphology (top) and pore size distribution (bottom) obtained by microtomography of cylindrical scaffolds with 3.5 mm of thickness and 15 mm of diameter. (**A**) PLA processed with Freon R134a; (**B**) PLA processed with scCO_2_ and (**C**) PLGA processed with Freon R134a. Scale bar 5 mm.

The microtomography analysis confirmed the heterogeneous pore size distributions in the three fabricated 3D structures, which were especially wide when Freon R134a was employed as the processing media. Indeed, the PLA and PLGA scaffolds processed with Freon R134a showed a heterogeneous pore size distribution, with high proportions of pores larger than 500 μm (Figure 4). Conversely, PLA processed with scCO_2_ exhibited a more homogeneous pore size distribution, with a high proportion of 50–500-μm pores. The impact of pressure on the diffusivity is linked to the plasticization effect and hydrostatic pressure, which is a key factor at higher pressure because it decreases the available free volume in the polymer mixture leading to a reduced diffusion coefficient. However, the plasticizing effect of the foaming agent at lower pressure is the key factor because it increases the polymer chain mobility, which in turn results in a higher diffusion coefficient [49,50]. 

A valuable processing aid in any gas foaming process is the plasticization of the polymer brought about by the dissolved blowing agent, which could induce a significant reduction in the melting and glass transition temperatures. In the case of CO_2_, this effect is well-reported [9]. Taking into account these considerations, the larger pore size and the larger total porosity of PLA achieved with Freon R134a at low pressure, compared with that achieved by scCO_2_ processing, is likely due to the larger Tm reduction and larger plasticization of the PLA upon exposure to Freon R134a. This could also be linked to the hydrostatic pressure under high pressure scCO_2_ processing, which might cause a decrease in gas diffusivity due to the reduction in the available free volume in the system.

Given that the minimum pore size that allows cell growth within scaffolds is approximately 150 μm [51,52,53] and that bone regeneration is enhanced with pore sizes of approximately 300 μm and higher [54], the pore structures obtained by the mild and environmentally friendly processing with Freon R143a seem to be adequate for tissue engineering applications.

To further characterize the scaffolds porosity and pore size, mercury intrusion may be considered. Due to the high pressures applied to the materials under study, however, mercury intrusion is not recommended when working with non-rigid materials, like those in this study, because of potential pore re-arrangement [55,56]. Thus, as detailed in the Methods section, the porosity of the three different materials was finally evaluated through the absolute density of unfoamed disks, the apparent density of foamed disks and the geometric densities as determined by helium pycnometry (Table 2 and Supplementary Materials (Figure S4)). Open porosity values were not directly measured. Instead, they were calculated by subtracting the closed porosity from the total porosity, which explains their relatively high errors compared with those of Pclosed and PT. As expected, the three tested materials were found to be highly porous, with values >80% in terms of total porosity. PLGA-based scaffolds presented the highest degree of total porosity, while the lowest corresponded to the PLA-based scaffolds processed with scCO_2_. However, large percentages of closed pores (>80%) were obtained in the PLA and PLGA scaffolds produced with Freon R134a, because of the gas foaming technique. In contrast, the PLA-based scaffolds processed with scCO_2_ exhibited lower closed porosity values (56.9%) and, therefore, a higher volume of open pores. At the explored processing conditions, the pore size was larger in the Freon R134a-processed materials, although scCO_2_ led to larger degrees of internal connectivity.

**Figure 5:**
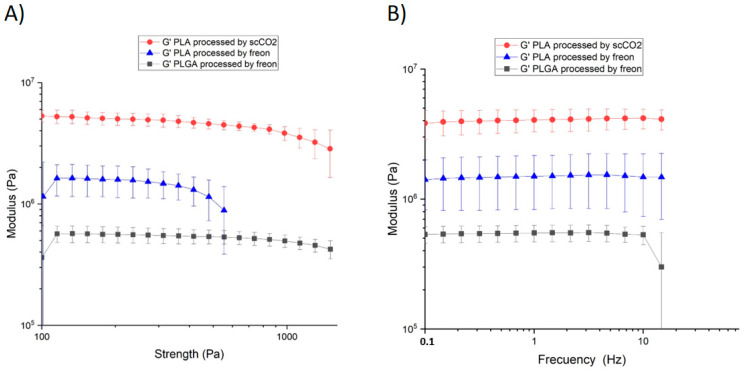
(**A**) Strain and (**B**) frequency sweeps of PLA processed with Freon R134a (blue triangles) or scCO_2_ (red dots), as well as PLGA processed with Freon R134a (grey squares).

Although a high degree of porosity, as well as large and open pores, are generally desired to facilitate cell ingrowth and vascularization, these characteristics might result in the scaffolds’ loss of mechanical properties due to the increased void volume, compromising their structural integrity. We performed a rheological analysis of the scaffolds to characterize their structural integrity by determining the dynamic (G’) and loss modulus (G″) in the linear-viscoelastic regime (Figure 5 and Figure S5). Strain sweeps were performed at a constant frequency of 1.0 Hz, and the pressure was varied from 100 Pa to 2000 Pa to study the range in which the scaffolds show a constant G’. We then fixed a value of 500 Pa and performed frequency sweeps from 0.1 Hz to 100 Hz. All scaffolds showed a linear G’ behavior from 100 Pa to 1500 Pa, except PLA processed with Freon R134a, whose linear range was 100–550 Pa (Figure 5A). The frequency varied from 0.1 Hz to 15 Hz for all samples (Figure 5B). The modulus achieved showed that the PLA processed with scCO_2_ was the hardest and most compact scaffold, reaching stability at 4.52 ± 0.58 MPa, followed by PLA processed with Freon R134a, which is also hard (G’ = 1.08 ± 0.49 MPa) within a similar range. The morphology, degree of heterogeneity and orientation of the pores, as well as the pore size distribution and open porosity, strongly influence the scaffold’s mechanical behavior. In this regard, differences in the shear moduli of PLA processed with CO_2_ and Freon134a can be related to their differences in porosity and pore size and distribution. As expected, the PLGA scaffold was the softest (G’ = 0.52 ± 0.08 MPa), resulting in a relatively fragile structure (see Supplementary Materials, Figure S5). Bone shows a complex, anisotropic structure in which highly different types of non-homogeneously distributed organic and inorganic matter are present. In addition, changes related to anatomical location, shape and physiopathological conditions result in major variations of the measured mechanical parameters, with the shear modulus values of trabecular bone ranging from 8 to 40 MPa [57]. Although the G’ values of the PLA scaffolds processed with scCO_2_ and FreonR134 are close to those measured in bone, these scaffolds might not meet the mechanical requirements for repairing load-bearing defects. However, there might be a window of opportunity in the treatment of non-load-bearing defects (e.g., for repairing craniofacial defects). Reinforced porous matrices could be obtained by preparing composites of polymer and bioceramic fillers. In fact, supercritical CO_2_ foaming allowed for the manufacture of porous composites of PLA and hydroxyapatite or β-TCP microparticles with superior viscoelastic properties [58]. 

### 3.3. Surface Functionalization of 3D Porous Scaffolds with GPF-Based pNPs

We next attempted to decorate the PLA scaffolds processed with Freon R134a or scCO_2_ with pNPs that promote cell growth [43]. GFP-based pNPs were adsorbed on the surface of the inner and external porous walls by means of a filtration step, and their localization was observed by confocal microscopy taking advantage of the pNP fluorescence, confirming that the internal connectivity of the pores created with Freon R134a or scCO_2_ allowed pNPs to reach the inner pores of the scaffold (Supplementary Materials, Figure S6). However, we observed slightly higher loading of pNPs in the scaffolds generated with scCO_2_ than with Freon R134a, both on the surface and inside the material. Quantification of retained pNPs in the functionalized scaffolds revealed that 69.8 ± 3.8% and 75.4 ± 2.2% of the loaded pNPs were adsorbed into the PLA scaffolds processed with Freon R134a and scCO_2_, respectively.

### 3.4. Cytocompatibility of 3D Porous Scaffolds

**Figure 6:**
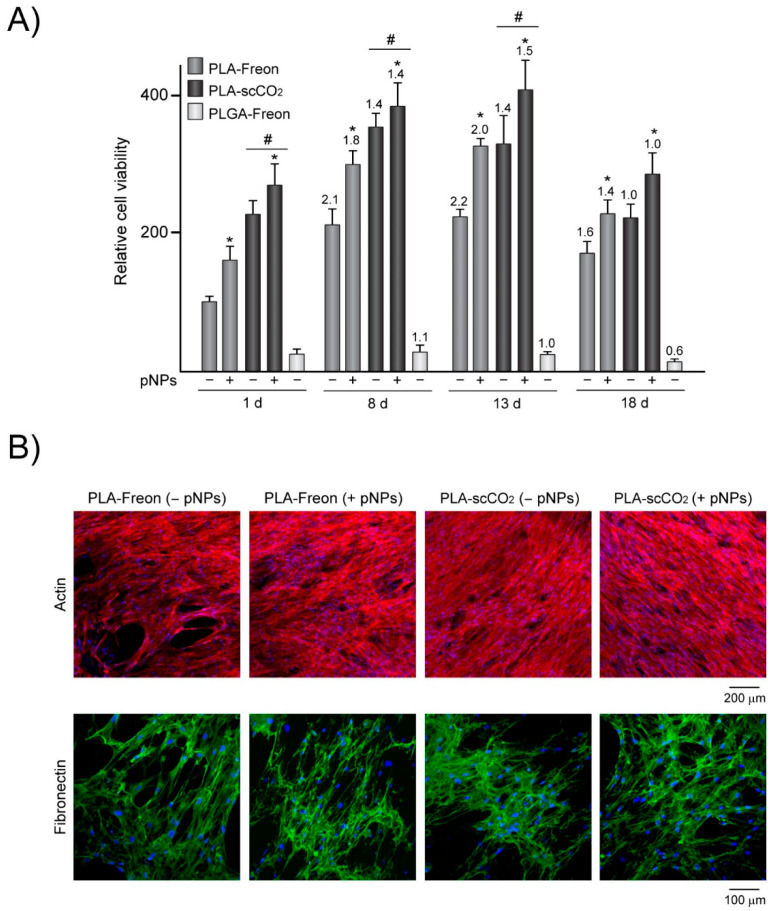
(**A**) Cell viability of MSCs cultured for 1–18 days on PLA processed with Freon R134a (PLA-Freon) or scCO_2_ (PLA-scCO_2_), decorated (+) or not (−) with pNPs, or on PLGA processed with Freon R134a (PLGA-Freon). The data were normalized relative to those measured in MSCs cultured for 1 day on PLA-Freon without pNPs, which were assigned an arbitrary value of 100. Fold-change values relative to cell viability at day 1 are shown above the graph bars. (**B**) Confocal maximum projections showing nuclei (blue), actin (red) or fibronectin (green) of MSCs cultured on the scaffolds for 8 days. * *p* < 0.05 compared with the scaffolds without pNPs. # *p* < 0.05 compared with PLA-Freon.

To evaluate the cytocompatibility of the PLA and PLGA scaffolds processed with Freon R134a, we cultured MSCs on these materials for 1–18 days (Figure 6). The metabolic activity data after culturing for one day showed that both polymeric matrices support MSC attachment, as observed in previous studies in which this cell type was cultured on polymeric scaffolds developed by conventional techniques [59,60]. The composition of the polymer has been shown to influence the scaffold’s structure [61]. In fact, processing of PLA and PLGA polymers with Freon R134a led to the generation of scaffolds with different structures, which might affect MSC behavior. The MSCs’ metabolic activity at day 1 was notably higher on the PLA scaffolds than on the PLGA scaffolds. The MSC viability on the PLGA scaffolds also did not increase over culture time (Figure 6A), suggesting that the physicochemical properties of PLGA scaffolds, including sharp edges (Figure 3C) and fragile microstructure (Figure S2), were inadequate for supporting MSC attachment and growth. In contrast to that observed for PLGA, the physicochemical properties of the PLA scaffolds processed with Freon R134a provided a more suitable environment for cell growth. A comparison between the PLA scaffolds processed with Freon R134a and with scCO_2_ revealed that the metabolic activity of the MSCs cultured for 1 day was reduced on the matrices processed with Freon R134a, which could be attributed to lower cell adhesion. The morphological features of the porous interfaces and, in particular, the pore sizes have an important effect on the number of cells that can attach and adhere to the materials. Regardless of the biomaterial or adherent cells tested, seeding efficiency decreases as the pore size increases [62,63,64]. Increased pore size reduces the surface area within the structure, lowering the available space for cell adhesion and limiting the cell attachment sites. In our study, MSCs likely attached at a higher extent to the surface of the PLA scaffolds processed with scCO_2_, with a higher proportion of 50–500-μm pores, than to scaffolds processed with Freon R134a, with a higher proportion of pores >500 μm (Figure 4). In addition to increased pore size, the PLA scaffolds processed with Freon R134a exhibited topographical features consisting of curved surfaces limited by sharp ridges (Figure 3), which could hinder the formation of focal adhesions, structures providing cell anchoring to attachment sites in the substrate. MSC viability increased from day 1 to day 8 on the PLA scaffolds processed with Freon R134a, and the increase was more pronounced than that observed on the matrices processed with scCO_2_. The metabolic activity of the MSCs cultured on the PLA scaffolds remained constant at day 13 and decreased at day 18. The microscopic examination revealed that the MSCs cultured for 8 days reached confluence on the PLA scaffolds processed with Freon R134a or scCO_2_ and displayed spread morphology with a similarly well-organized actin cytoskeleton and a dense matrix of fibronectin fibrils (Figure 6B). Lastly, we studied the impact of functionalized PLA scaffolds with pNPs [27,28,65]. There were no signs of toxicity promoted by pNPs in the cells cultured on the pNP-decorated scaffolds, as revealed by alamarBlue assay and DAPI staining. On the contrary, the functionalization of the PLA scaffolds processed with Freon R134a or scCO_2_ improved MSC viability, an effect observed at all studied periods. High tunability regarding protein composition, as well as the architectural features of pNPs, provides a huge range of possibilities for combining mechanical, biochemical and temporal stimulation of selected cellular responses such as adhesion, proliferation and differentiation [43,66,67]. 

Although the pore interconnectivity and open porosity of the fabricated structures is low, open porosity is expected to increase as the scaffold degrades, facilitating cell migration to the inside. Scaffolds processed with Freon R134a show a high percentage of pores larger than 500 μm (Figure 4) and with a wall width between pores much smaller than the pore size, as clearly observed in the SEM images of longitudinal and cross-sections of the processed polymers (Figure 3). The features of Freon R134a-processed PLA are ideal for the rapid formation of open porosity, which depends on the rate of polymer degradation and on the microscopic structural characteristics of the separation between the scaffold’s pores. 

## 4. Conclusions

Scaffolds consisting of PLA can be successfully engineered by compressed fluid technology using dense Freon R134a as a solvent media, obtaining desirable structural characteristics such as a high degree of total porosity (>80%) with high proportions of pores larger than 500 μm and a G’ of 1.08 ± 0.49 MPa. This manufacturing method is performed at a working pressure of 2 MPa, nearly one order of magnitude lower than that employed in scaffold manufacturing with scCO_2_ (>10 MPa). CO_2_ is a gas at 2 MPa and above 25 °C; therefore, polymer foaming at such low pressures cannot be achieved with this compressible gas. This reduced operating pressure will have a positive impact in terms of reduced equipment and operational costs and increased safety and on process scalability of industrially relevant foaming technologies, such as extrusion foaming and bead foaming, performed up to now with scCO_2_ at higher pressures. 

In this study, we produced for the first time polymeric porous materials for biomedical applications using dense Freon R134a as a solvent media and demonstrated its processing advantages, which could be further improved upon through technological development. Further research on optimizing the processing parameters such as the working pressure and depressurization flow, which have been shown to have a significant impact on CO_2_-processed polymeric scaffolds and could likely lead to enhanced results in terms of a higher percentage of open porosity with dense Freon R134a and even higher cytocompatibility. The presented method could also be employed for polymers in which the compressed gas can diffuse and plasticize. Freon R134a has been shown to be a good plasticizer, and it can solubilize in polyvinyl acetate, cellulosic polymers, and certain vinylidene fluoride polymers, in addition to saturated aliphatic polyesters, such as PLA and PLGA, thereby promising a wide applicability of the methodology reported herein [68]. In vitro studies using human MSCs have shown that PLA scaffolds processed with Freon R134a are cytocompatible. This study also demonstrates for the first time that pNP functionalization of PLA scaffolds processed with Freon R134a is an effective approach for increasing the viability of osteoprogenitor cells, taking into account its processing advantages. 

## Figures and Tables

**Table 1:** Experimental conditions for preparing the porous polymeric matrices.

Stage	Processing Parameters	Material				
		PLA	PLA	PLA	PLA	PLGA
**Polymer disk preparation**	**Thermal annealing**	Yes	Yes	Yes	Yes	No
	**m (g)**	0.4	0.4	0.8	0.8	0.3
	**T (°C)**	150	150	150	150	-
	**Applied pressure (Kg)**	3000	3000	3000	3000	500
**Porous scaffold fabrication**	**Compressed fluid**	Freon R134a	Freon R134a	scCO_2_	scCO_2_	Freon R134a
	**Tw (°C)**	40	40	35	35	35
	**Pw (MPa)**	2	2	10.3	10.3	2
	**Soaking time, t (h)**	3	3	2	2	2
**pNP Scaffold decoration**	**-**	No	Yes	No	Yes	No

**Table 2:** Porosities of the studied 3D porous scaffolds.

Material	Total Porosity, P_T_ (%)	Closed Porosity, P_closed_ (%)	Open Porosity, P_O_ (%)
PLA-Freon R134a	92.8 ± 2.0	83.2 ± 3.3	9.6 ± 1.3
PLA-scCO_2_	82.3 ± 0.9	56.9 ± 7.5	25.4 ± 6.6
PLGA-Freon R134a	96.7 ± 0.8	89.1 ± 3.3	7.6 ± 3.4

Mean porosity values and the corresponding standard deviations were calculated, as described in Methods section, from experimental measurements performed over 3 samples of each type of scaffold.

## Data Availability

The data presented in this study are available on request from the corresponding author.

## Supplementary Materials

The following are available online at https://www.mdpi.com/article/10.3390/polym13203453/s1, Figure S1: DSC thermogram of raw PLA before annealing, Figure S2: DSC thermogram of PLA after annealing at 150 °C, Figure S3: Picture of a PLA scaffold with a non-complete foaming visually observed at the center of the disk., Figure S4: Absolute and apparent densities of the studied porous scaffolds, Figure S5: (A) Strain and (B) frequency sweeps of PLA processed with Freon R-134a (blue) and scCO2 (red) as well as PLGA processed with Freon R-134a (grey squares), Figure S6: pNPs penetrability in PLA-based scaffolds.
