# Supplementary material for: Polylactide, Processed by a Foaming Method Using Compressed Freon R134a, for Tissue Engineering
Source: Polymers (Basel). 2021 Oct 9;13(20):3453. doi: 10.3390/polym13203453 (PMC8539307; doi:10.3390/polym13203453)
Supplement: Supplementary file 1 [file polymers-13-03453-s001.zip › polymers-1386582-SM.pdf]

polymers

Supplementary Materials

Polylactide, Processed by a Foaming Method Using Compressed Freon R134a, for Tissue Engineering

María Aguado [1,2], Laura Saldaña [2,3], Eduardo Pérez del Río [1,2], Judith Guasch [1,2,4], Marc Parera [1,2], Alba Córdoba [1,2], Joaquín Seras-Franzoso [2,4,5], Olivia Cano-Garrido [2,4,5], Esther Vázquez [2,4,5], Antonio Villaverde [2,4,5], Jaume Veciana [1,2], Imma Ratera [1,2,*], Nuria Vilaboa [2,3,*] and Nora Ventosa [1,2,*]

[1] Institut de Ciència de Materials de Barcelona, ICMAB-CSIC, Campus UAB, 08193 Bellaterra, Spain; m.aguado.olalla@gmail.com (M.A.); eperez2@icmab.es (E.P.d.R.); jguasch@icmab.es (J.G.); marc.parera@applus.com (M.P.); acordoba@nanomol-tech.com (A.C.); vecianaj@icmab.es (J.V.)
[2] CIBER de Bioingeniería, Biomateriales y Nanomedicina (CIBER-BBN), 28029 Madrid, Spain; laura.saldana@salud.madrid.org (L.S.); joaquin.seras@gmail.com (J.S.-F.); olivia.cano.garrido@gmail.com (O.C.-G.); Esther.Vazquez@uab.cat (E.V.); antoni.villaverde@uab.cat (A.V.)
[3] Hospital Universitario La Paz-IdiPAZ, Paseo de la Castellana 261, 28046 Madrid, Spain
[4] Dynamic Biomimetics for Cancer Immunotherapy, Max Planck Partner Group, ICMAB-CSIC, Campus UAB, Bellaterra, 08193 Barcelona, Spain
[5] Institut de Biotecnologia i Biomedicina, Universitat Autònoma de Barcelona, Bellaterra, 08193 Barcelona, Spain
[6] Departament de Genètica i de Microbiologia, Universitat Autònoma de Barcelona, Bellaterra, 08193 Barcelona, Spain
* Correspondence: iratera@icmab.es (I.R.); nuria.vilaboa@salud.madrid.org (N.Vi.); ventosa@icmab.es (N.Ve.)

Citation: Aguado, M.; Saldaña, L.; del Río, E.; Guasch, J.; Parera, M.; Córdoba, A.; Seras-Franzoso, J.; Cano-Garrido, O.; Vázquez, E.; Villaverde, A.; et al. Polylactide, Processed by a Foaming Method Using Compressed Freon R134a, for Tissue Engineering. *Polymers* **2021**, *13*, 3453. https://doi.org/10.3390/polym13203453

Academic Editor(s): José Miguel Ferri; Vicent Fombuena Borràs; Miguel Fernando Aldás Carrasco

Received: 2 September 2021
Accepted: 1 October 2021
Published: 9 October 2021

Publisher's Note: MDPI stays neutral with regard to jurisdictional claims in published maps and institutional affiliations.

Copyright: © 2021 by the authors. Submitted for possible open access publication under the terms and conditions of the Creative Commons Attribution (CC BY) license (https://creativecommons.org/licenses/by/4.0/).

The DSC thermograms below show how the melting peak observed around 110ºC for the non-treated PLA sample (Figure S4) disappears after the thermal annealing of the disk at 150ºC (Figure S5), indicating that the material is amorphysed.

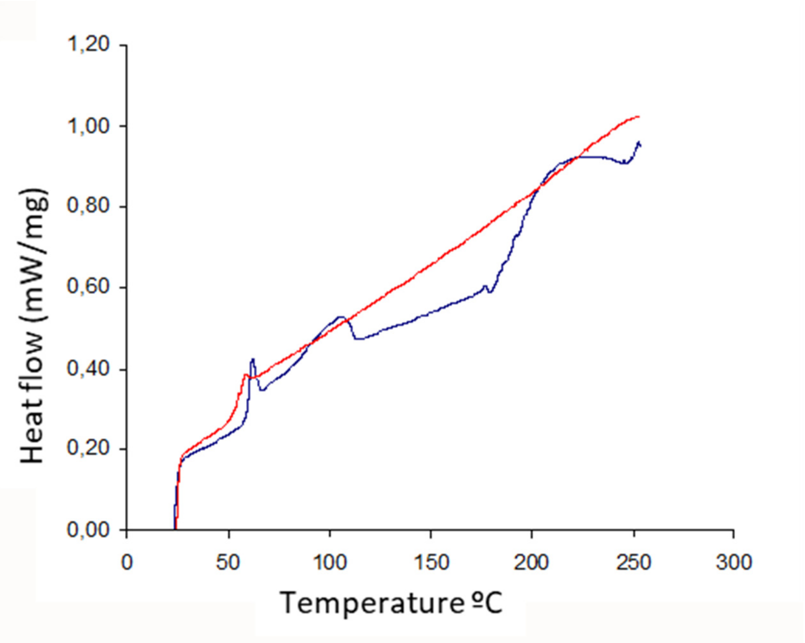

Figure S1. DSC thermogram of raw PLA before annealing. Blue: first heating cycle; Red: second heating cycle.

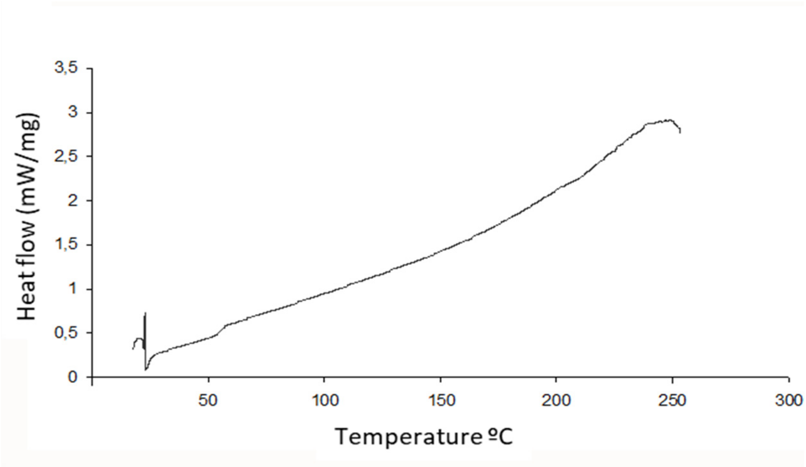

Figure S2. DSC thermogram of PLA after annealing at 150ºC.

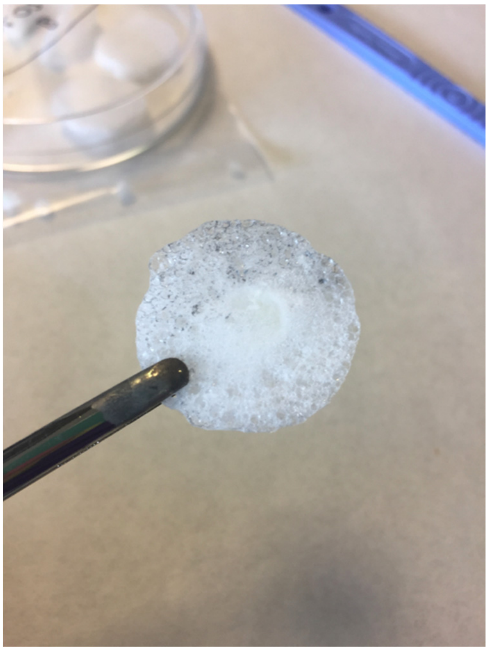

Figure S3. Non-complete foaming of PLA was visually observed at the center of the disk.

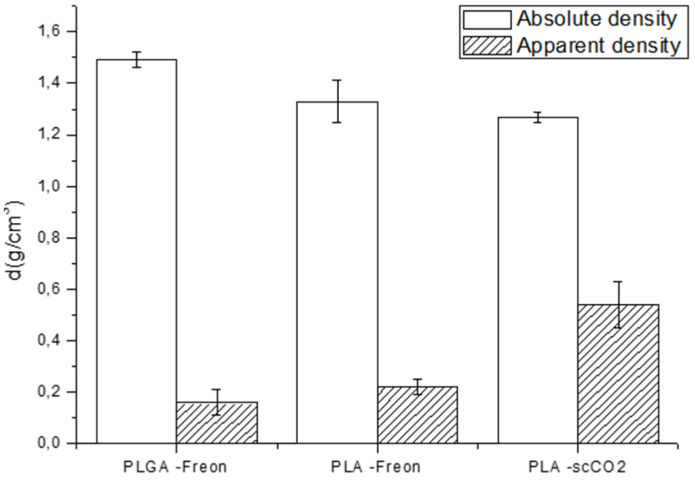

Figure S4. Absolute and apparent densities of the studied porous scaffolds. The values shown correspond to the mean values of three specimens ± standard error.

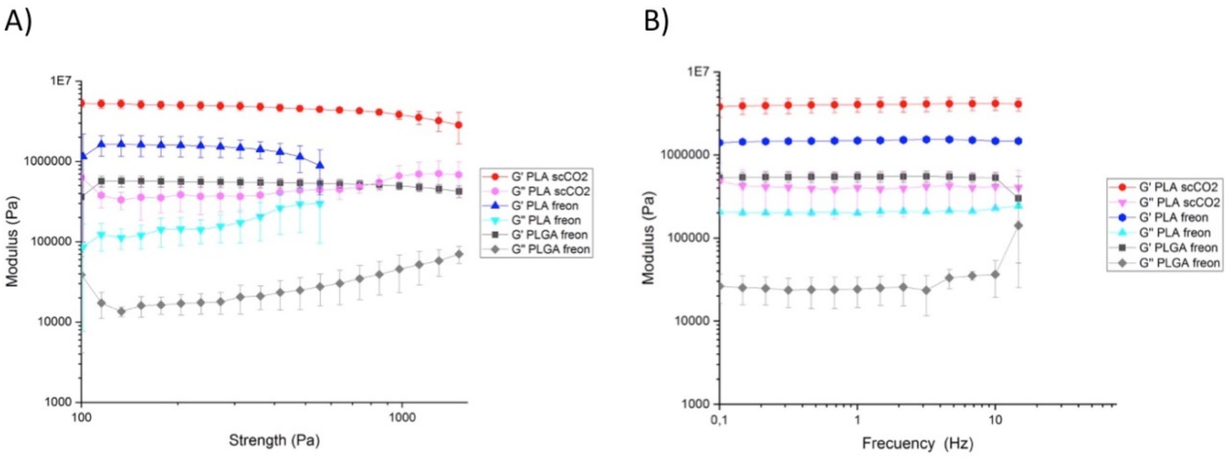

Figure S5. A) Strain and B) frequency sweeps of PLA processed with Freon R-134a (blue) and $scCO_2$ (red) as well as PLGA processed with Freon R-134a (grey squares).

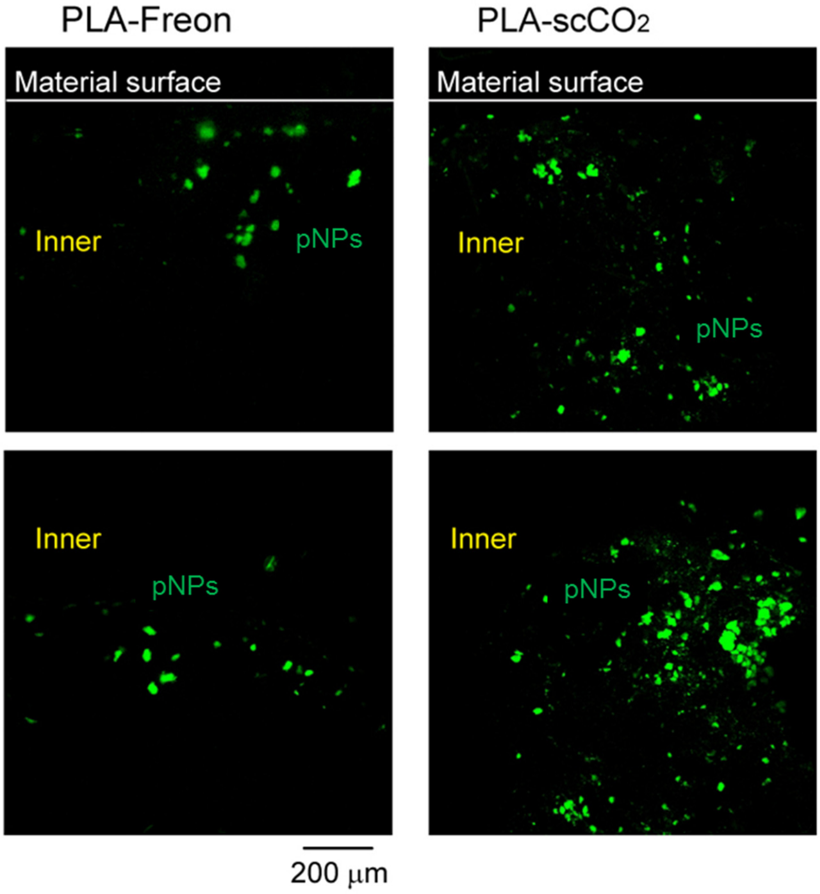

Figure S6. pNPs penetrability in PLA-based scaffolds. Confocal microscopy images corresponding to cross section of PLA processed with Freon (PLA-Freon) and PLA processed with $scCO_2$ (PLA-$scCO_2$) decorated with pNPs. The white line marks the border of the material surface and the inner part of scaffold.
